# A systematic review and meta-analysis of glucocorticoids treatment in severe COVID-19: methylprednisolone versus dexamethasone

**DOI:** 10.1186/s12879-023-08280-2

**Published:** 2023-05-05

**Authors:** Shukun Hong, Hongye Wang, Shuyuan Li, Jian Liu, Lujun Qiao

**Affiliations:** 1 Department of Intensive Care Unit, Shengli Oilfield Central Hospital, Dongying, China; 2 Department of Obstetrics and Gynecology, Shengli Oilfield Central Hospital, Dongying, China

**Keywords:** Methylprednisolone, Dexamethasone, Glucocorticoids, COVID-19, Meta-analysis

## Abstract

**Objective:**

The preferred agent of glucocorticoids in the treatment of patients with severe COVID-19 is still controversial. This study aimed to compare the efficacy and safety of methylprednisolone and dexamethasone in the treatment of patients with severe COVID-19.

**Methods:**

By searching the electronic literature database including PubMed, Cochrane Central Register of Controlled Trials, and Web of Science, the clinical studies comparing methylprednisolone and dexamethasone in the treatment of severe COVID-19 were selected according to the inclusion criteria and exclusion criteria. Relevant data were extracted and literature quality was assessed. The primary outcome was short-term mortality. The secondary outcomes were the rates of ICU admission and mechanical ventilation, PaO_2_/FiO_2_ ratio, plasma levels of C-reactive protein (CRP), ferritin, and neutrophil/lymphocyte ratio, hospital stay, and the incidence of severe adverse events. Statistical pooling applied the fixed or random effects model and reported as risk ratio (RR) or mean difference (MD) with the corresponding 95% confidence interval (CI). Meta-analysis was performed using Review Manager 5.1.0.

**Results:**

Twelve clinical studies were eligible, including three randomized controlled trials (RCTs) and nine non-RCTs. A total of 2506 patients with COVID-19 were analyzed, of which 1242 (49.6%) received methylprednisolone and 1264 (50.4%) received dexamethasone treatment. In general, the heterogeneity across studies was significant, and the equivalent doses of methylprednisolone were higher than that of dexamethasone. Our meta-analysis showed that methylprednisolone treatment in severe COVID-19 patients was related to significantly reduced plasma ferritin and neutrophil/lymphocyte ratio compared with dexamethasone, and that no significant difference in other clinical outcomes between the two groups was found. However, subgroup analyses of RCTs demonstrated that methylprednisolone treatment was associated with reduced short-term mortality, and decreased CRP level compared with dexamethasone. Moreover, subgroup analyses observed that severe COVID-19 patients treated with a moderate dose (2 mg/kg/day) of methylprednisolone were related to a better prognosis than those treated with dexamethasone.

**Conclusions:**

This study showed that compared with dexamethasone, methylprednisolone could reduce the systemic inflammatory response in severe COVID-19, and its effect was equivalent to that of dexamethasone on other clinical outcomes. It should be noted that the equivalent dose of methylprednisolone used was higher. Based on the evidence of subgroup analyses of RCTs, methylprednisolone, preferably at a moderate dose, has an advantage over dexamethasone in the treatment of patients with severe COVID-19.

**Supplementary Information:**

The online version contains supplementary material available at 10.1186/s12879-023-08280-2.

## Introduction

The coronavirus disease 2019 (COVID-19) caused by Severe Acute Respiratory Syndrome Coronavirus 2 (SARS-CoV-2), is considered to be the third outbreak of β coronaviruses in the twentieth and twenty-first centuries, after SARS-CoV and Middle East respiratory syndrome coronavirus (MERS-CoV) [1]. As of 13 November 2022, 632 million confirmed COVID-19 cases and 6.5 million deaths have been reported globally [2]. Approximately 5% of patients with COVID-19 need transferring to the intensive care unit (ICU) for respiratory support and are associated with high mortality [3, 4]. The therapeutic drugs investigated for COVID -19, such as hydroxychloroquine [5], lopinavir/ritonavir [6], azithromycin [7], and ivermectin [8], could not bring definitely favorable clinical effects.

The pathophysiology of severe COVID-19 involves a host-mediated inflammatory response that leads to serious endothelial injury and alveolar damage [4, 9]. Many studies showed that patients with severe COVID-19 tended to have a higher level of inflammatory cytokines, including white blood cells, neutrophils, procalcitonin, C-reactive protein (CRP), interleukin, ferritin, and so on [3, 10, 11]. To prevent tissue damage caused by an excessive inflammatory response, glucocorticoid, as the classic anti-inflammatory drug, has been rapidly applied. The RECOVERY trial conducted in the UK has demonstrated that dexamethasone can significantly decrease mortality in cases with severe COVID-19, especially in patients receiving mechanical ventilation support, as compared to standard care without corticosteroids [12]. A multicenter randomized controlled trial (RCT) conducted by Villar et al. found that dexamethasone could reduce overall mortality and duration of mechanical ventilation in COVID-19 patients with moderate-to-severe acute respiratory distress syndrome (ARDS) [13]. Based on these studies, World Health Organization (WHO) recommends the use of systemic corticosteroids in patients with severe COVID-19 [14].

However, it is well known that the preferred glucocorticoid for the treatment of ARDS in ICU is methylprednisolone rather than dexamethasone. Several studies have revealed that compared with no glucocorticoid treatment, methylprednisolone also showed a mortality advantage in the treatment of severe COVID-19 [15–17]. Some researchers have compared the clinical effects of the two glucocorticoids during the current epidemic [18–29]. Nevertheless, the results are inconsistent.

The purpose of this meta-analysis was to compare the efficacy and safety of methylprednisolone and dexamethasone in the treatment of patients with severe COVID-19, and to provide evidence-based proof for the optimal management of COVID-19 pneumonia.

## Materials and methods

This study was carried out in line with the Statement of Preferred Reporting Items for Systematic Reviews and Meta-Analyses (PRISMA) [30]. All stages of literature search, study selection, data extraction, and quality assessment were performed independently by two authors. Any disagreements between the two authors were resolved by discussion or arbitration by a third author.

### Search strategy

The literature search was performed as we described previously [17]. The following electronic databases were searched: PubMed, Cochrane Central Register of Controlled Trials, and Web of Science. The following search strategy was used in PubMed and changes depending on the rules of each database: (COVID-19) AND (((corticosteroids) OR (methylprednisolone)) OR (dexamethasone)). The latest search was conducted on 10 August 2022. No language or geographical restrictions were applied during literature searches. All references cited in the relevant articles were screened to identify eligible studies.

### Study selection

Studies comparing methylprednisolone with dexamethasone in the treatment of severe COVID-19 met the inclusion criteria of our meta-analysis. According to the guideline specified by the Chinese National Health Commission [23], severe COVID-19 is defined as meeting any of the following items: ①Respiratory rate ≥ 30 breaths per minute; ②Oxygen saturation ≤ 93% on room air at rest; ③Arterial oxygen pressure / inspired oxygen fraction ≤ 300 mmHg; ④Clinical symptoms are gradually aggravated, and the pulmonary imaging shows that the lesions progress more than 50% within 24–48 h. In cases of duplicates, the most recent or the most complete publication was used. Studies comparing methylprednisolone or dexamethasone with no corticosteroid therapy for patients with COVID-19 were excluded. Reviews, case reports, letters, editorials, and comparative studies which presented insufficient data were excluded.

### Data extraction and quality assessment

The following information was extracted using standardized data extraction forms for each study: the first author’s last name; year of publication; study design; country; study interval; sample size, gender composition, mean age, intervention method in each group; inclusion criteria, primary outcome and other study features and data needed for quality assessment. Mean and standard deviation were used for extracting continuous variables. If studies reported continuous data as median and/or range values, the standard deviation was calculated using statistical algorithms by Hozo et al. [31]. The number of events and the total number of participants in each group were used for extracting binary variables. The primary outcome of our meta-analysis was short-term mortality, which involves in-hospital, 28-day, 30-day and 50-day mortality corresponding to the definition used in each study. The secondary outcomes included the rates of ICU admission and mechanical ventilation, PaO_2_/FiO_2_ ratio, systemic inflammatory markers (CRP, ferritin, and neutrophil/lymphocyte ratio), hospital stay, and the incidence of severe adverse events. The methodological quality of the RCTs was assessed according to the criteria specified by the Cochrane Collaboration [32]. For non-RCTs, the methodological index for non-randomized studies (MINORS) was used for quality assessment [33].

### Statistical analysis

The outcomes were pooled as an estimate of the overall effect for the meta-analysis conducted using Review Manage, version 5.1.0 (The Cochrane Collaboration, 2011). As we previously reported [17, 34], for dichotomous variables, the pooled risk ratio (RR) with corresponding 95% confidence interval (CI) was aggregated in Mantel–Haenszel method, and the mean difference (MD) with corresponding 95% CI was calculated in inverse variance method for continuous variables. Clinical heterogeneity was discussed when appropriate. Subgroup analysis was performed on the basis of different types of study design, the daily dosage of methylprednisolone, and the treatment course of corticosteroids. Statistical heterogeneity was assessed by Cochran’s *Q* test and *I*^*2*^ statistic with *p* < 0.1 or *I*^*2*^ > 50% considered as significant. An *I*^*2*^ value of 0% indicates no observed statistical heterogeneity. If the statistical heterogeneity was not significant, then the fixed-effect model was used; otherwise, the random effects model would be applied. Sensitivity analysis was carried out, if applicable, by excluding studies to remove heterogeneity when it was statistically significant. A forest plot was constructed to graphically assess the statistical heterogeneity by displaying effect estimates and 95% CI for both individual studies and meta-analyses. The *p* value threshold for statistical significance was set at 0.05 for effect sizes. Publication bias was explored by Begg’s funnel plot and Egger’s regression test with *p* < 0.05 considered as significant (STATA 12.0).

## Results

### Study selection

**Fig. 1:**
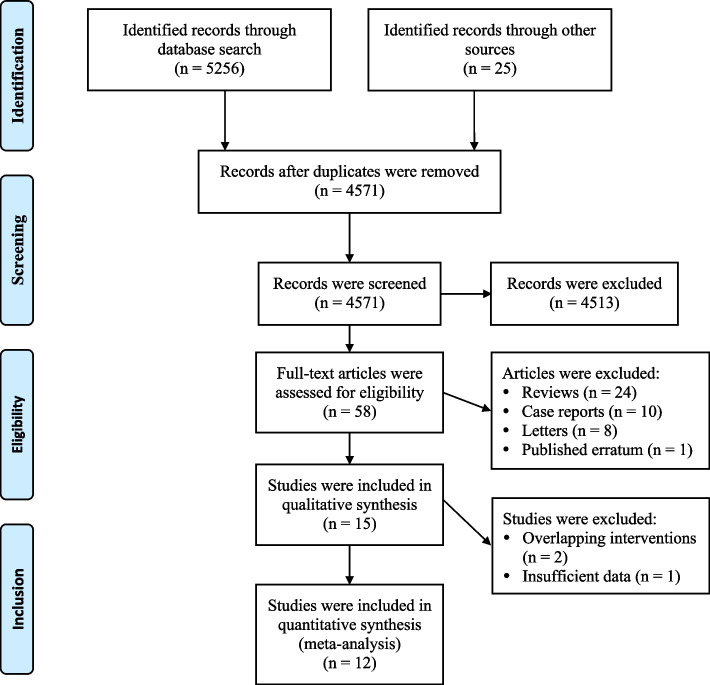
Study flow diagram chart

The flowchart of study selection is presented in Fig. 1. Overall, we identified 5281 citations by the initial literature searches. Among them, 710 were removed as duplicates, and 4513 were excluded after titles and abstracts screening. We retrieved 58 full-text articles for detailed evaluation. After reviewing, 43 references were excluded for the following reasons: reviews (*n* = 24), case reports (*n* = 10), letters (*n* = 8), and published erratum (*n* = 1). Then, 15 studies were included for qualitative synthesis. Of them, two studies [35, 36] in which the methylprednisolone group involved dexamethasone treatment were further excluded, and one study [37] providing insufficient data was also excluded. Finally, 9 non-RCTs [18–26] and 3 RCTs [27–29] matched the inclusion criteria and were suitable for our meta-analysis. A total of 2506 patients with severe COVID-19 were analyzed, of which 1242 (49.6%) received methylprednisolone and 1264 (50.4%) received dexamethasone treatment.

### Study characteristics

The characteristics of the twelve included studies are summarized in Table 1. Three studies were conducted in Pakistan [18–20], two in Egypt [28, 29], and the remaining were respectively carried out from Italy [21], South Africa [22], Morocco [23], USA [24], Spain [25], Colombia [26] and Iran [27]. The study interval in each study ranged from March 2020 to October 2021. The percentage of females in each arm ranged from 24.0% to 53.3%, without significant difference between the groups (RR 0.89; 95% CI 0.75–1.04; *p* = 0.14). The mean age of the patients varied between 52 years and 74.4 years across the studies and was not significantly different between the two groups (MD -0.16 years; 95% CI − 1.47 to 1.15; *p* = 0.81). As shown in Table 1, the severity of COVID-19 patients included in all studies was at least severe. The doses and treatment courses of corticosteroids were not consistent across the studies, and the equivalent doses of methylprednisolone were higher than that of dexamethasone.

**Table 1:** The characteristics of the included studies

Author, year	Study design / country	Study interval	Sample size (F / M)		Mean age		Inclusion criteria	Intervention	Primary outcome	Quality assessment ^a^
			MG	DG	MG	DG				
Fatima, 2020 [18]	Multicenter, non-RCT / Pakistan	1 June 2020 to 30 June 2020	65 (NA)	35 (NA)	54.86	57.91	1. Age 18–75 years 2. COVID-19 PCR positive 3. O_2_ saturation < 94% on room air, regardless of chest X-ray findings 4. Moderate or severe COVID-19 disease according to operational definition 5.Informed consent obtained	MG: 1 mg/kg/day in 2 divided doses for 5 days DG: 8 mg/day for 5 days	NA	17
Rana, 2020 [19]	Single-center, non-RCT / Pakistan	NA	30 (9 / 21)	30 (10 / 20)	53.9	53.8	1. COVID-19 patients 2. Ventilated in ICU / high-dependency unit	MG: 40 mg twice daily for 8 days DG: 8 mg twice daily for 8 days	PaO_2_/FiO_2_ ratio	13
Aslam, 2021 [20]	Single-center, non-RCT / Pakistan	May 2021 to June 2021	41 (NA)	41 (NA)	NA		1. Severe COVID-19 pneumonia 2. Admitted in high-dependency unit	MG: 1–2 mg/kg/day DG: 6–8 mg/day	Decrease or increase in oxygen demand	16
Buso, 2021 [21]	Single-center, non-RCT / Italy	1 December 2020 to 31 January 2021	136 (44 / 92)	110 (36 / 74)	74.4	72.1	1. Adult patients 2. COVID-19 PCR positive 3. Presence of pneumonia detected using imaging 4. Supplementary oxygen	MG: 60 mg/day for ⩾10 days, then tapered gradually DG: 6 mg/day for ⩾10 days, then tapered gradually	30-day all-cause mortality and the need for semi-ICU or ICU admission	17
Du Plessis, 2021 [22]	Single-center, non-RCT / South Africa	26 March 2020 to 18 July 2020	46 (14 / 32)	108 (52 / 56)	52	54.5	1. Severe COVID-19 pneumonia 2. Admitted to a COVID-dedicated ICU for respiratory support	MG: 80 mg/day for 10–14 days DG: 8 mg/day for 10–14 days	NA	15
El mezzeoui, 2021 [23]	Single-center, non-RCT / Morocco	1 March 2020 to 30 December 2020	230 (68 / 162)	283 (100 / 183)	64	63	1. Over 18 years of age 2. COVID-19 PCR positive or Chest CT typical 3. ICU stay ⩾ 7 days	MG: 1 mg/kg/day for 7 days DG: 6 mg/day for 7 days	Improvement in clinical and biological parameters	15
Ko, 2021 [24]	Single-center, non-RCT / USA	1 March 2020 to 31 July 2020	104 (25 / 79)	83 (21 / 62)	56.2	57.8	1. COVID-19 PCR positive 2. Admitted to ICU for respiratory failure from COVID-19 3. Oxygen requirement of ⩾40 L/min and FiO_2_ of ⩾50%	MG: 1 mg/kg/day for ⩾3 days DG: 6 mg/day for ⩾7 days	50-day all-cause mortality	17
Mora-Luján, 2021 [25]	Single-center, non-RCT / Spain	March 2020 to April 2021	189 (49 / 140)	199 (56 / 143)	67	67.8	1. COVID-19 PCR positive 2. Supplementary oxygen 3. Presenting with elevated inflammatory parameters	MG: ⩾100 mg/day for 3 days DG: 6 mg/day for 10 days	In-hospital mortality	18
Pinzón, 2021 [26]	Single-center, non-RCT / Colombia	11 June 2020 to 31 October 2020	105 (38 / 67)	111 (51 / 60)	64	63	1. Over 18 years of age 2. COVID-19 PCR positive 3. Radiological confirmation of pneumonia by chest CT 4. Supplementary oxygen	MG: 250–500 mg/day for 3 days, followed by prednisone 50 mg/day orally for 14 days DG: 6 mg/day for 7–10 days	Recovery time	13
Ranjbar, 2021 [27]	Single-center, RCT / Iran	August 2020 to November 2020	44 (17 / 27)	42 (20 / 22)	56.2	61.3	1. Over 18 years of age 2. COVID-19 PCR positive 3. O_2_ saturation < 92% on room air	MG: 2 mg/kg/day, and tapered to half dosage every 5 days DG: 6 mg/day for 10 days	28-day all-cause mortality and clinical status after 5 as well as 10 days after enrollment with 9-point WHO ordinal scale	Low risk
Saeed, 2022 [28]	Single-center,RCT / Egypt	June 2020 to October 2021	222 (NA)	192 (NA)	NA		1. Over 18 years of age 2. COVID-19 PCR positive 3. Ventilated patients with ARDS	MG: 2 mg/kg/day for 5 days, then tapered to half dosage for another 5 days DG: 6 mg/day for 10 days	Clinical, laboratory, and radiological status after 10 days of enrollment	Low risk
Soliman, 2022 [29]	Single-center,RCT / Egypt	NA	30 (13 / 17)	30 (16 / 14)	60.6	58.13	1. Over 18 years of age 2. COVID-19 patients with destructive inflammatory immune response 3. Needing ICU admission	MG: 1 mg/kg/day for 7 days DG: 8 mg/day for 7 days	Inflammatory response monitoring by neutrophil / lymphocyte ratio	Low risk

*F / M* Female / Male, *MG* Methylprednisolone group, *DG* Dexamethasone group, *RCT* Randomized controlled trial, *NA* Not available

^a^ Methodological index for non-randomized studies (MINORS) was used for quality assessment of non-RCT, risk of bias was used for quality assessment of RCT

### Quality assessment and publication bias

**Fig. 2:**
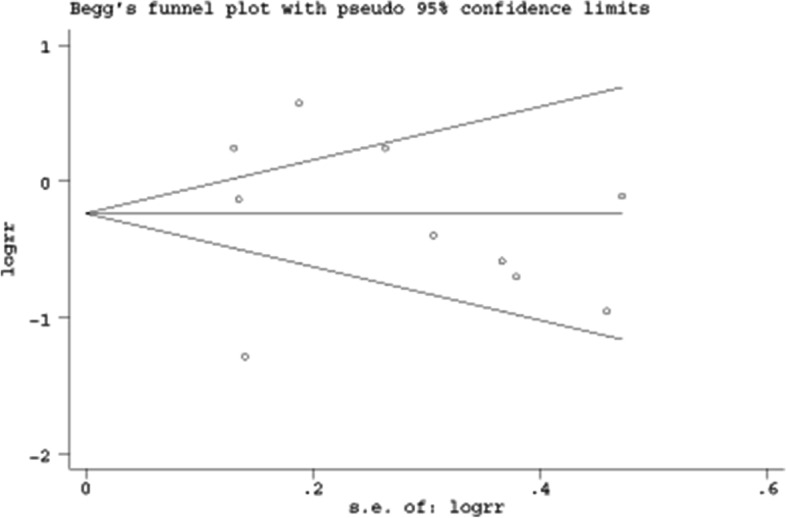
Begg’s funnel plot for publication bias

**Fig. 3:**
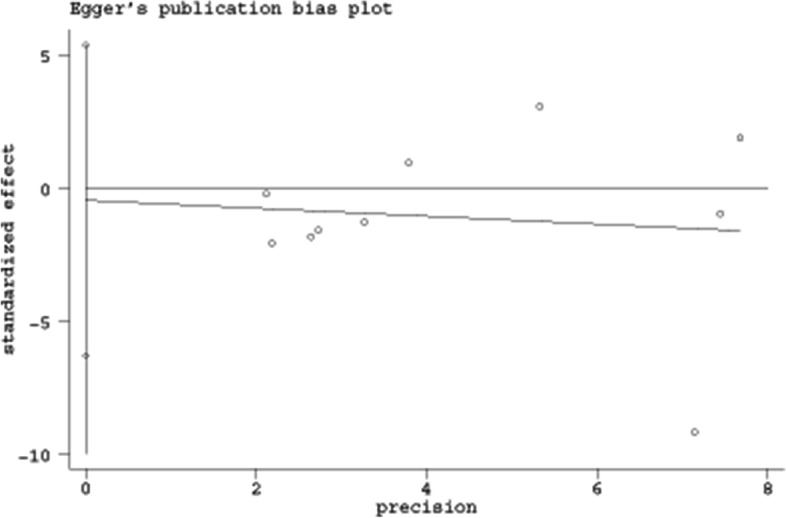
Egger’s regression analysis for publication bias

The methodological quality assessments of the included literature are briefly described in Table 1, and separately summarized in Supplementary Table 1 and Supplementary Fig. 1. Based on the MINORS scoring system, the scores of 9 non-RCTs ranged from 13 to 18 points. According to the criteria of risk of bias, the 3 RCTs were classified as low risk. Overall, the included studies were of moderate to high quality. Begg’s funnel plot constructed for the visual evaluation of publication bias revealed a slight asymmetry (Fig. 2). However, Egger’s regression test demonstrated that the visual asymmetry was not statistically significant (95% CI of intercept -6.32 to 5.31; *p* = 0.855) (Fig. 3).

### Primary outcome

**Fig. 4:**
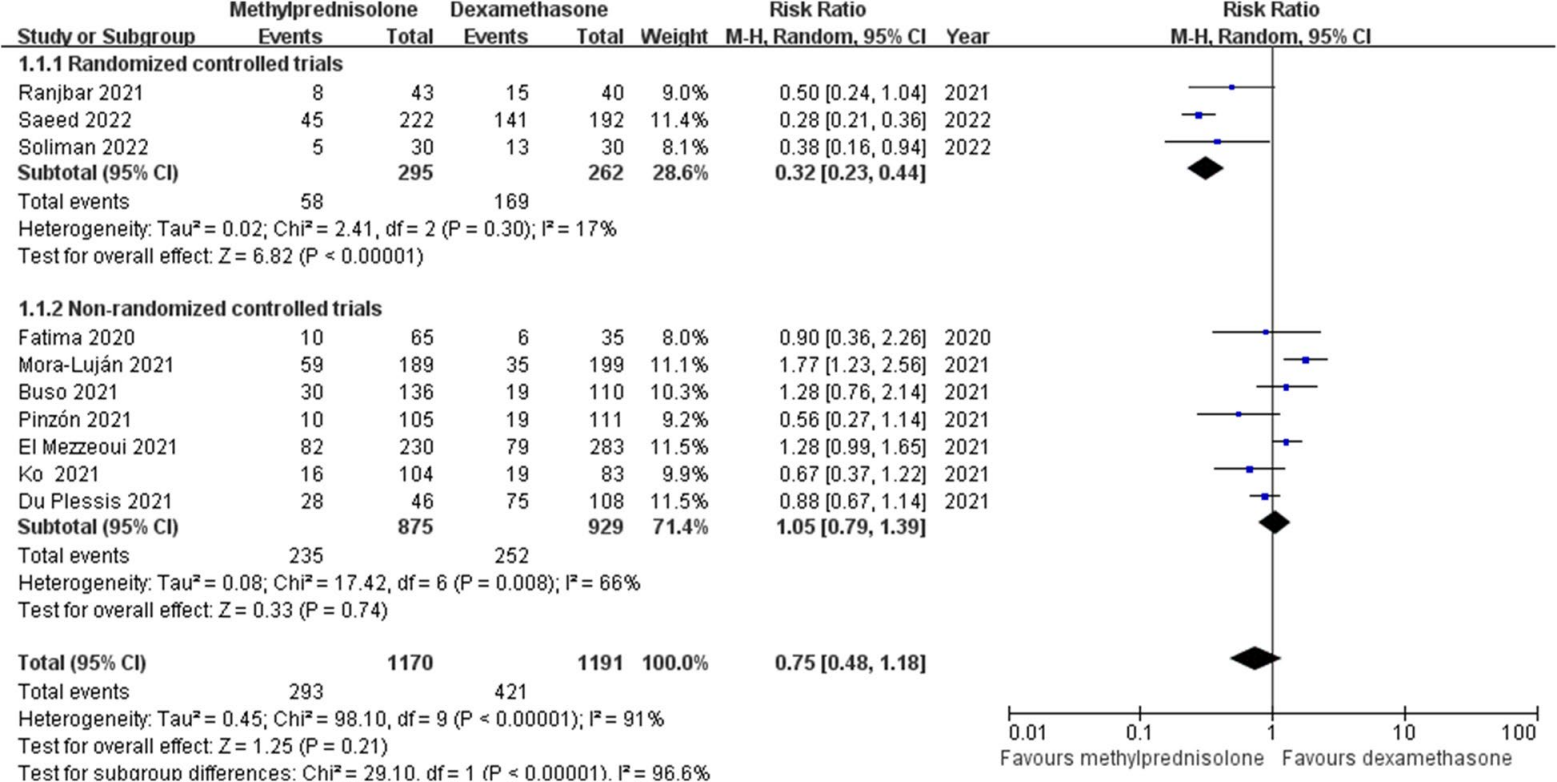
Forest plot of the short-term mortality after corticosteroids treatment

Ten included studies [18, 21–29] provided the data of short-term mortality after corticosteroids therapy. When these studies were pooled, the overall mortality was 30.2%. There were 293 deaths (25.0%) among 1170 patients receiving methylprednisolone and 421 deaths (35.3%) among 1191 patients receiving dexamethasone. Due to the significant heterogeneity across studies (*p* < 0.1, *I*^*2*^ = 91%), random effects model was applied. Our meta-analysis showed that the mortality was not significantly decreased in the methylprednisolone group compared with the dexamethasone group (RR 0.75; 95% CI 0.48–1.18; *p* = 0.21) (Fig. 4). This result was consistent with the subgroup analysis of non-RCTs, while in the subgroup analysis of RCTs, the mortality of the methylprednisolone group was significantly lower than that of the dexamethasone group (Fig. 4). In the subgroup analysis of shorter or longer courses of glucocorticoid treatment, the difference in mortality between methylprednisolone and dexamethasone was not significant (Supplementary Fig. 2). Besides, methylprednisolone at a dose of 1 mg/kg/day or > 2 mg/kg/day had the same effect on mortality as dexamethasone, while its dose of 2 mg/kg/day could significantly reduce the mortality of patients with severe COVID-19 (Supplementary Fig. 3).

### Secondary outcomes

#### ICU admission

**Fig. 5:**
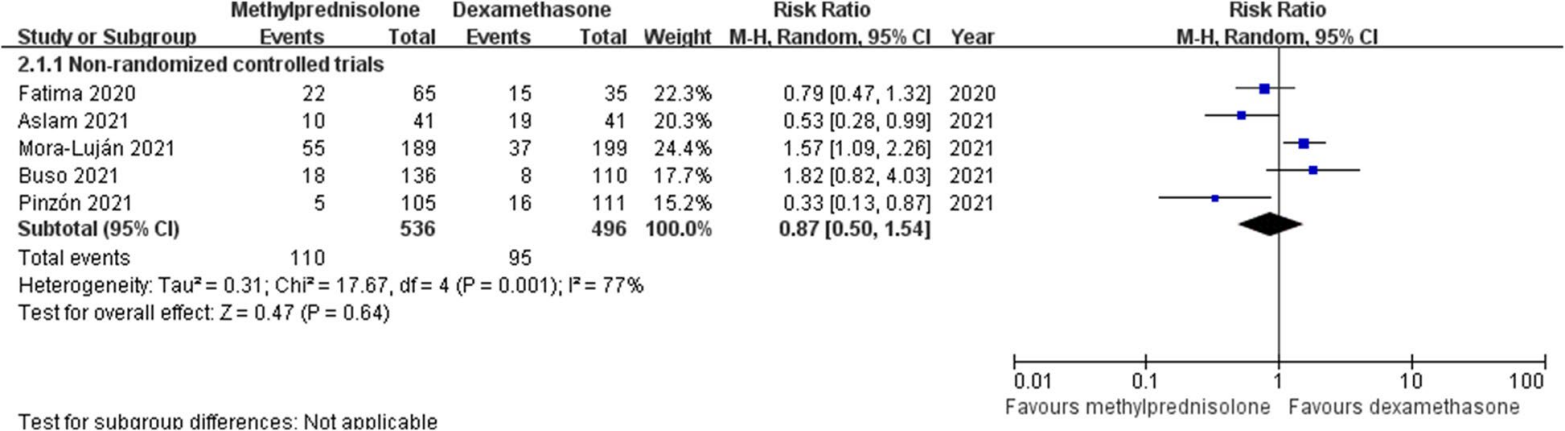
Forest plot of the ICU admission rate after corticosteroids treatment

There were five non-RCTs [18, 20, 21, 25, 26] reporting the information about ICU admission. Overall, the rate of ICU admission in this analysis was 19.9%, with 110 cases (20.5%) in the methylprednisolone group and 95 cases (19.2%) in the dexamethasone group. Random effects model was used owing to a significant heterogeneity across studies (*p* < 0.1, *I*^*2*^ = 77%). Our pooling results revealed that the difference in ICU admission rate between the two groups was not statistically significant (RR 0.87; 95% CI 0.50–1.54; *p* = 0.64) (Fig. 5). This result was in accordance with the outcomes of subgroup analyses on different courses of glucocorticoids treatment (Supplementary Fig. 4).

### Mechanical ventilation

**Fig. 6:**
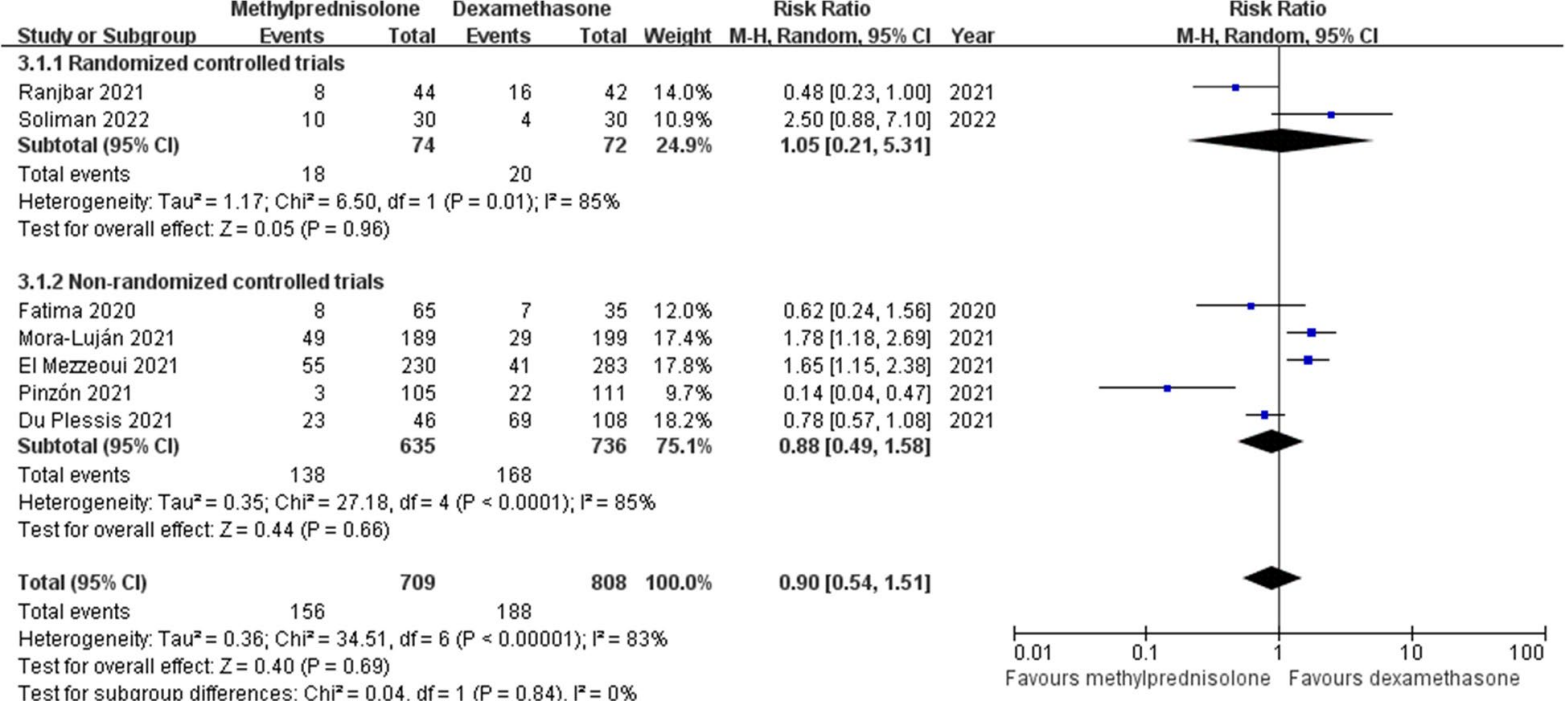
Forest plot of the need for mechanical ventilation after corticosteroids treatment

Information regarding the need for mechanical ventilation after corticosteroids administration was described in seven studies [18, 22, 23, 25–27, 29]. In this analysis, the general rate of mechanical ventilation in patients with severe COVID-19 was 22.7%. The number of patients receiving mechanical ventilation was 156 (22.0%) in the methylprednisolone group and 188 (23.3%) in the dexamethasone group, respectively. Random effects model was used to synthesize the data because of a significant heterogeneity among studies (*p* < 0.1, *I*^*2*^ = 83%). No significant difference in mechanical ventilation rate between the groups was detected in our meta-analysis (RR 0.90; 95% CI 0.54–1.51; *p* = 0.69) (Fig. 6) and subgroup analyses of different treatment courses (Supplementary Fig. 5).

### PaO_2_/FiO_2_ ratio

**Fig. 7:**
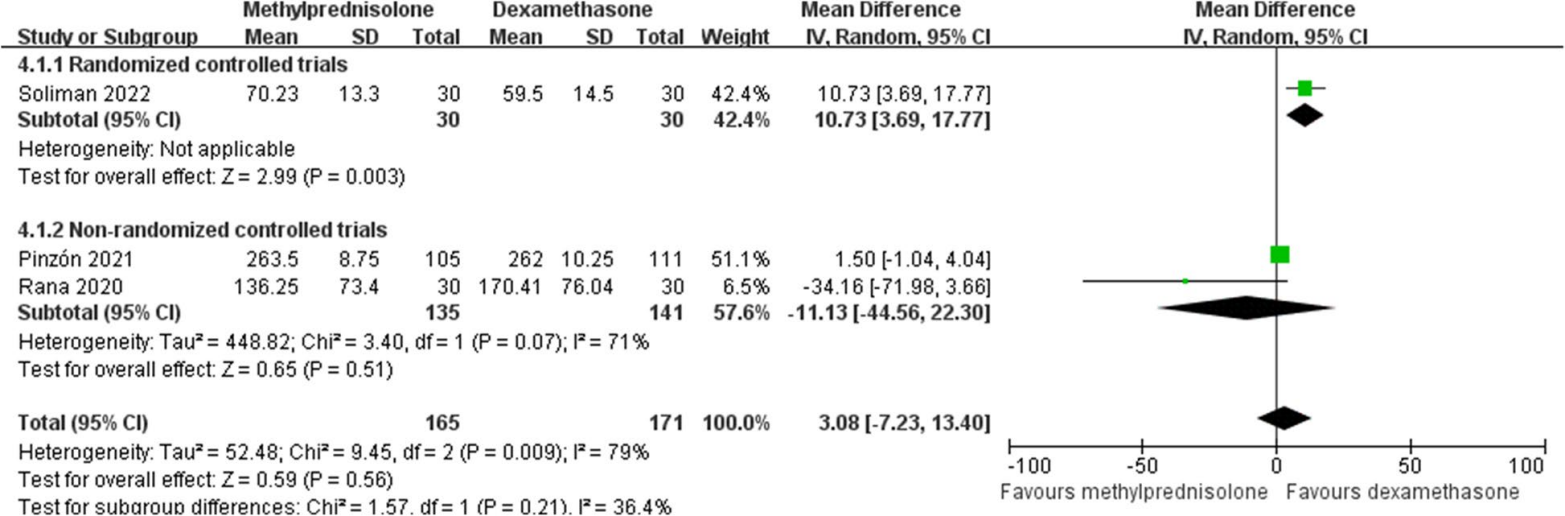
Forest plot of the PaO_2_/FiO_2_ ratio after corticosteroids treatment

There were three studies [19, 26, 29] recording the data of the PaO_2_/FiO_2_ ratio after corticosteroids treatment. Random effects model was employed since the heterogeneity among studies was significant (*p* < 0.1, *I*^*2*^ = 79%). The difference between the treatments in PaO_2_/FiO_2_ ratio was not statistically significant in our meta-analysis (MD 3.08; 95% CI -7.23 to 13.40; *p* = 0.56) (Fig. 7) and subgroup analysis of non-RCTs. Since only one RCT provided data, subgroup analysis for RCTs cannot be performed. However, this RCT [29] showed the benefit of methylprednisolone in increasing the PaO_2_/FiO_2_ ratio.

### Systemic inflammatory markers

**Fig. 8:**
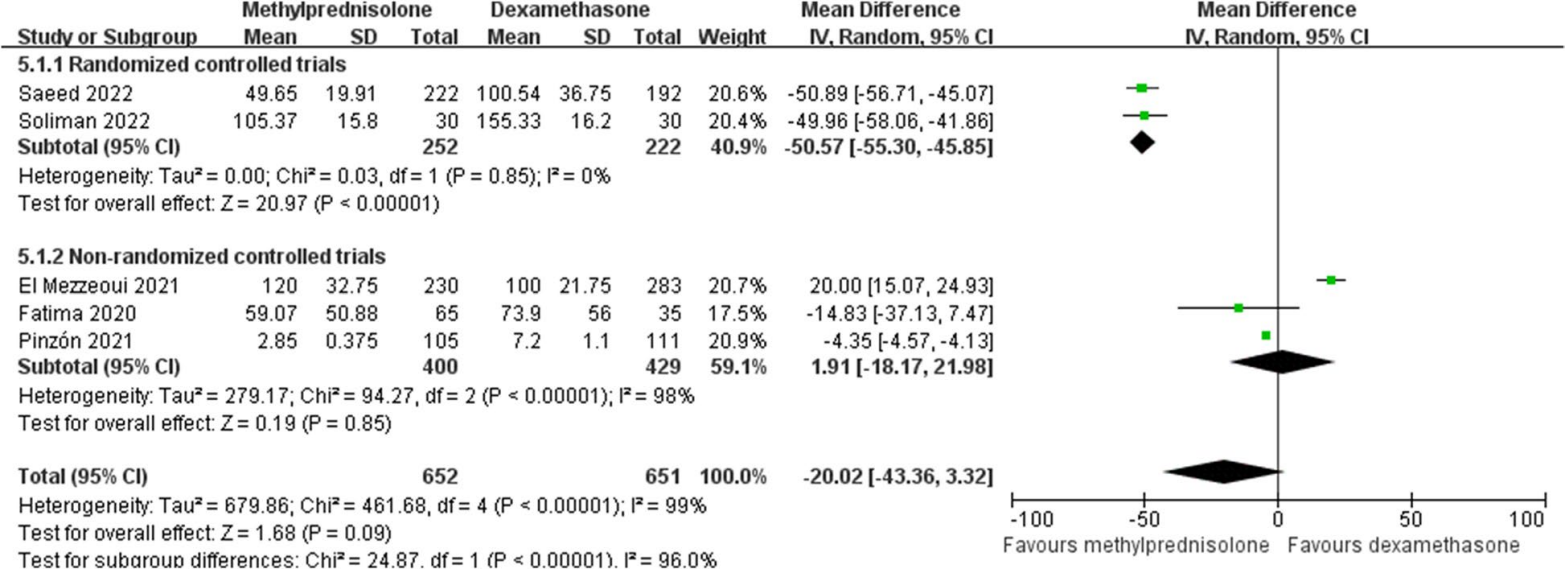
Forest plot of the plasma CRP level after corticosteroids treatment

Data about plasma CRP levels following corticosteroids application was collected from five studies [18, 23, 26, 28, 29]. As heterogeneity across studies was significant (*p* < 0.1, *I*^*2*^ = 99%), random effects model was adopted. Our meta-analysis found no significant difference in plasma CRP level between two groups (MD -20.02; 95% CI -43.36 to -3.32; *p* = 0.09) (Fig. 8). Nevertheless, subgroup analysis of RCTs showed that the plasma CRP level was significantly reduced in patients treated with methylprednisolone (Fig. 8).

**Fig. 9:**
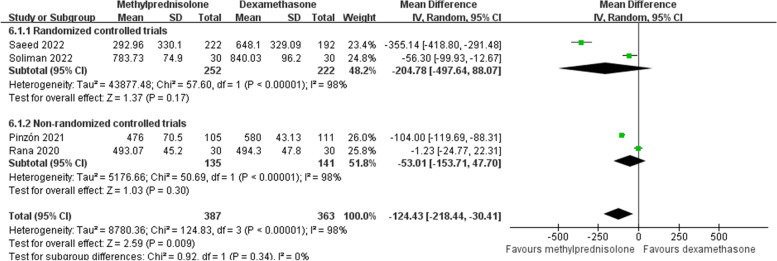
Forest plot of the plasma ferritin value after corticosteroids treatment

Four studies [19, 26, 28, 29] measured the plasma ferritin value after the intervention. Random effects model was chosen due to a significant heterogeneity among studies (*p* < 0.1, *I*^*2*^ = 98%). Meta-analysis indicated that the plasma ferritin value was significantly lower in the methylprednisolone group than in the dexamethasone group (MD -124.43; 95% CI -218.44 to -30.41; *p* = 0.009), although subgroup analyses of RCTs and non-RCTs both implied no significant difference between two groups in this outcome (Fig. 9).

**Fig. 10:**
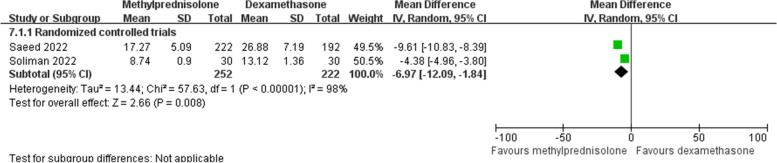
Forest plot of the neutrophil/lymphocyte ratio after corticosteroids treatment

Two included RCTs [28, 29] calculated the neutrophil/lymphocyte ratio after steroids treatment. Meta-analysis on random effects model demonstrated that this ratio was significantly reduced in patients receiving methylprednisolone than in those receiving dexamethasone (MD -6.97; 95% CI -12.09 to -1.84; *p* = 0.008) (Fig. 10).

### Hospital stay

**Fig. 11:**
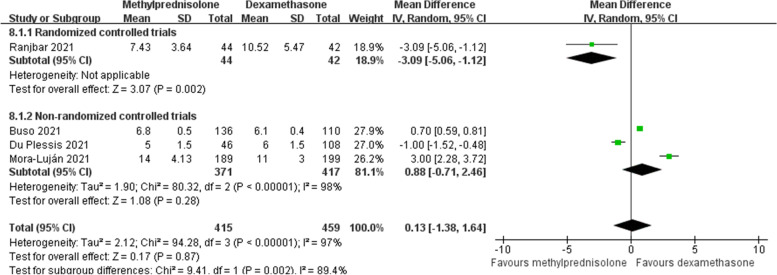
Forest plot of the hospital stay after corticosteroids treatment

There were four studies [21, 22, 25, 27] reporting the length of hospital stay. Owing to a significant heterogeneity among studies (*p* < 0.1, *I*^*2*^ = 97%), random effects model was used. Our meta-analysis observed that the length of hospital stay was not significantly different between the two groups (MD 0.13; 95% CI -1.38 to 1.64; *p* = 0.87) (Fig. 11). This finding was consistent with the result of subgroup analysis of non-RCT. Interestingly, an included RCT [27] supported the effect of methylprednisolone on shortening the length of hospitalization. However, due to the limited number of studies, subgroup analysis of RCTs cannot be conducted.

### Severe adverse events

**Fig. 12:**
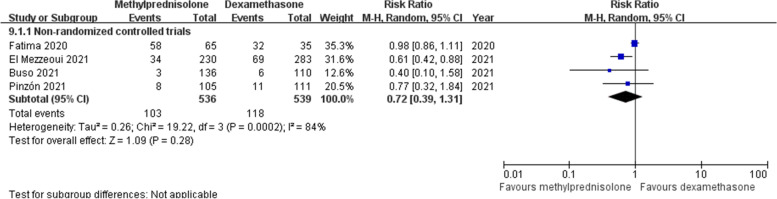
Forest plot of the incidence of hyperglycemia after corticosteroids treatment

Data about hyperglycemia after corticosteroids treatment was recorded in four non-RCTs [18, 21, 23, 26]. Meta-analysis on random effects model demonstrated that the incidences of hyperglycemia in the methylprednisolone group (19.2%) and the dexamethasone group (21.9%) were comparable (RR 0.72; 95% CI 0.39–1.31; *p* = 0.28) (Fig. 12).

**Fig. 13:**
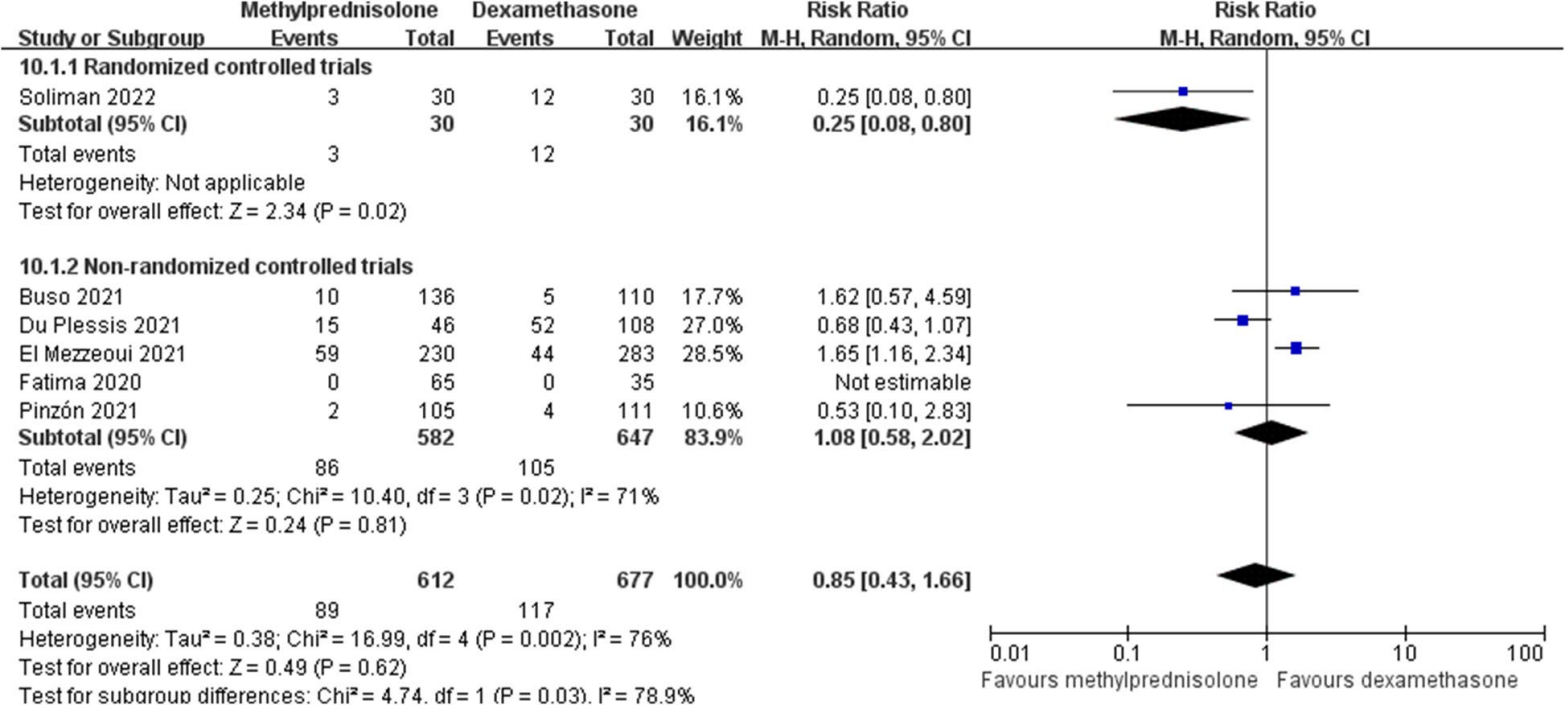
Forest plot of the incidence of nosocomial infections after corticosteroids treatment

In our analysis, there were six studies [18, 21–23, 26, 29] providing the data of nosocomial infections. Overall, 89 (14.5%) cases treated with methylprednisolone and 117 (17.3%) cases treated with dexamethasone suffered from nosocomial infections. Random effects model was utilized due to a significant heterogeneity across studies (*p* < 0.1, *I*^*2*^ = 71%). This meta-analysis failed to show the significant difference in the incidence of nosocomial infections between the two groups (RR 0.85; 95% CI 0.43–1.66; *p* = 0.62) (Fig. 13).

### Sensitivity analysis

We performed sensitivity analyses on all interested outcomes due to the significant heterogeneity among studies. However, sensitivity analyses did not change the significance of statistical heterogeneity and had no effect on the results of the meta-analysis, except for the incidence of hyperglycemia. After excluding the study by Fatima et al. [18], the statistical heterogeneity between studies was eliminated (*p* = 0.73, *I*^*2*^ = 0%). Sensitivity analysis indicated that the risk of hyperglycemia in the methylprednisolone group was significantly lower than that in the dexamethasone group (RR 0.61; 95% CI 0.44–0.85; *p* = 0.004) (Supplementary Fig. 6).

## Discussion

This meta-analysis including 12 clinical studies found that methylprednisolone could significantly reduce the systemic inflammatory response in severe COVID-19 patients, and its effect was equivalent to that of dexamethasone on other clinical outcomes. Nevertheless, subgroup analyses of RCTs demonstrated that methylprednisolone treatment in severe COVID-19 was associated with reduced short-term mortality compared with dexamethasone. Moreover, severe COVID-19 patients treated with a moderate dose (2 mg/kg/day) of methylprednisolone were related to a better prognosis than those treated with dexamethasone.

It is reported that excessive inflammatory reaction is closely related to the severity of patients with severe COVID-19. In brief, when the body suffers from hyper-inflammatory reaction, immune cells such as T cells, macrophages and natural killer cells are over activated and proliferated. These cells produce a large number of inflammatory mediators, which cause damage to lung epithelium and endothelial cells. The consequence of this pathological change is the occurrence of ARDS, which is the main cause of death of patients with COVID-19 [38–40]. Thereby, according to this theory, the comprehensive treatment strategy of COVID-19 should include measures to resist hyperinflammation.

Glucocorticoids can inhibit the transcription and secretion of various cytokines, reduce systemic inflammation and exudate in lung tissue, and minimize the risk of respiratory failure [41]. They have been used previously in respiratory diseases such as asthma, chronic obstructive pulmonary disease, severe bacterial pneumonia, and ARDS. During the SARS epidemic, Zhao et al. [42] conducted a comparative study and observed that early administration of high-dose methylprednisolone was associated with reduced mortality compared with other treatment regimens. However, this mortality benefit of glucocorticoids did not appear in the MERS epidemic. Arabi et al. [43] reported in their study that corticosteroid therapy in critically ill patients with MERS was not associated with lower 90-day mortality but was related to delayed MERS-CoV RNA clearance, as compared to no corticosteroid therapy. Nevertheless, the mortality advantage of glucocorticoids has been found again in the current epidemic of coronavirus pneumonia [12]. A meta-analysis collected data from 7 randomized clinical trials to evaluate the effectiveness of glucocorticoids in 1703 critically ill patients with COVID-19 [44]. The results showed that administration of systemic corticosteroids was associated with lower 28-day all-cause mortality compared with usual care or placebo. Another meta-analysis including 44 studies and 20,197 patients with COVID-19 also demonstrated a beneficial effect of corticosteroids on short-term mortality [45].

Although this beneficial effect of glucocorticoids in severe COVID-19 is confirmed by WHO [14], it is still unclear which glucocorticoid drug is preferred. Some scholars suggest that corticosteroids in general are not expected to help as a class of drugs, but rather each steroid should be assessed individually because different drugs can be associated with a different number of genes [46]. Many published meta-analyses have evaluated the role of glucocorticoids in the COVID-19 epidemic, but they have not evaluated the comparison between glucocorticoids [45, 47–51].

The glucocorticoids used in the reported studies are mainly methylprednisolone and dexamethasone. For the treatment of severe patients, the National Health Commission of the China suggests methylprednisolone 0.5–1 mg/kg/day for 3–5 days [52], while the WHO and the National Institutes of Health of the United States recommend the use of dexamethasone 6 mg/day for 7–10 days [14, 53]. Buso et al. retrospectively analyzed 246 COVID-19 patients who needed oxygen supplementation and had received glucocorticoids therapy. They found that the choice of different glucocorticoids did not affect the main clinical outcomes [21]. In contrast, Ko et al. compared the effectiveness of methylprednisolone to dexamethasone in patients requiring intensive care, and demonstrated a mortality benefit in the methylprednisolone group [24]. This finding is consistent with the results of our subgroup analysis of RCTs, although our meta-analysis failed to confirm such mortality benefit of methylprednisolone. However, our meta-analysis observed that methylprednisolone was more effective in reducing the systemic inflammatory response than dexamethasone.

The reasons for such different clinical outcomes may be related to the inflammatory pathways and characteristics of the different glucocorticoids. Generally, the therapeutic effects of glucocorticoids can be mediated through genomic and more rapid-onset non-genomic mechanisms [54]. A study by Draghici et al. [46] described an initial characterization of the main pro-inflammatory pathways induced by SARS-Cov-2 infection on human lung epithelial cells, and detected methylprednisolone as the most effective agent that targets critical components of the inflammatory pathway responsible for ARDS. The study also suggested that methylprednisolone would revert the largest number of the gene perturbed by COVID-19, followed by dexamethasone. The non-genomic mechanism is dose-dependent with glucocorticoids, and the response of methylprednisolone was higher than that of dexamethasone in vitro [55, 56]. In addition, methylprednisolone could obtain a higher lung tissue-to-plasma ratio than dexamethasone in the animal model [57, 58]. From the perspective of mechanism, the combination of the above factors may make methylprednisolone more effective in reducing hyper-inflammation of the lung. In that case, the reduction of inflammation may lead to the rapid improvement of lung injury and easier relief of symptoms. The decrease of inflammatory markers found in this meta-analysis and the improvement of PaO_2_/FiO_2_ ratio and hospital stay shown in subgroup analyses just prove the advantage of methylprednisolone. These may partly explain the potential mortality advantage of methylprednisolone in the treatment of severe patients with COVID-19 [17]. Interestingly, we noticed in the subgroup analysis that such an advantage of methylprednisolone was associated with its dose of 2 mg/kg/day. We speculate that this may be related to the aforementioned dose-dependent characteristics of the hormones. Perhaps this moderate dose is the optimal dose of methylprednisolone for severe COVID-19 treatment.

It should be noted that the doses of the two glucocorticoids used in the included studies are not equivalent. The daily dose of methylprednisolone was at least 60 mg, if calculated according to the adult weight of 60 kg. In contrast, the dose of dexamethasone was 6–8 mg, equivalent to 32–42 mg of methylprednisolone. One may point out that this advantage of methylprednisolone is due to its high equivalent dose. However, there is no direct data in our meta-analysis to prove that the difference of outcomes is completely caused by the difference between the two hormone doses. The dose–effect of a hormone may be easily assessed, while the dose–effect between different types of hormones cannot be evaluated, because the factor in the characteristics of hormones cannot be completely excluded. We suggest that this issue needs further research.

When therapeutic drugs achieve good efficacy, safety is also a significant consideration. One of the adverse events of glucocorticoids treatment is hyperglycemia. Glucocorticoids can promote lipolysis, increase liver glucose output and raise insulin resistance, leading to elevation of blood glucose. In addition, patients receiving glucocorticoids therapy may also suffer from fluid retention, adrenal suppression, and secondary bacterial and fungal infections [54, 59]. In our meta-analysis, no difference was detected in the incidences of hyperglycemia and nosocomial infections between the two glucocorticoid treatments. Nevertheless, the results from sensitivity analysis suggest that methylprednisolone is safer because the incidences of adverse events in this group were significantly lower than that in the dexamethasone group.

It has been reported that the use of glucocorticoids would delay viral clearance in patients with COVID-19 [60, 61]. However, evidence from a recently published meta-analysis showed that low-dose corticosteroids did not have a significant impact on the duration of SARS-CoV-2 viral shedding [62]. The retrospective study by Buso et al. discovered no significant difference in virus clearance between the two glucocorticoids, while the time of virus clearance in the methylprednisolone group tended to be relatively shorter [21]. Although our meta-analysis showed a benefit of methylprednisolone in reducing inflammatory markers, whether it has an impact on virus clearance at high equivalent doses remains unresolved. To say the least, even if glucocorticoids are not conducive to virus clearance, their potential survival benefit in severe COVID-19 will outweigh the risk of prolonged viral shedding.

It is worth noting that the safety of glucocorticoids discussed in this meta-analysis is only for ordinary adult patients with COVID-19, but not for some special populations, such as pregnant women, because there is a lack of research in such groups that requires more strict ethics and highly careful study design [63].

Coincidentally, some systematic reviews [64–66] on the same topic have been published recently. One review [65] published in the form of "Letters" also included the three RCTs analyzed in our study. The information provided in that article was relatively incomplete and only three outcomes were analyzed. The authors of that article carried out another review [66] on non-RCTs, however, the number of included studies was relatively small. The evidence in the present study is significantly different from that in these reviews. In contrast, the number of included studies and the total sample size in our meta-analysis are larger. We believe that the larger the sample size, the lower the probability of false-negative results, and the more reliable the conclusions. Moreover, our meta-analysis investigated more interested outcomes, which is more meaningful for clinical guidance.

There are limitations to the present meta-analysis which need to be mentioned. First, the number of RCTs included in the meta-analysis is relatively restricted. We are aware that the pooling of data from non-RCTs is a debated topic in the field of meta-analysis as it may exaggerate the effect magnitude of an intervention. However, during the current COVID-19 epidemic, RCTs comparing methylprednisolone and dexamethasone in the treatment of severe COVID-19 are scarce. Many people are still infected and die every day, which creates a great threat to human society. In order to find valuable evidence-based proof in the treatment of severe COVID-19 as soon as possible, we collected non-RCTs for meta-analysis to increase the sample size as much as possible. In this way, the possibility of false-negative results can be minimized. Second, we are aware that there is a significant clinical heterogeneity across the included studies. Clinical factors such as the study design, the inconsistent inclusion criteria, and the dosage and course of glucocorticoids treatment of each study may have inordinately influenced the results of this systematic analysis. Given this consideration, we chose the random effects model for data synthesis and performed subgroup analyses to minimize the interference of these factors. Third, the meta-analysis of partial outcomes is based on a limited number of studies, which may reduce the power of the results. However, this does not mean that these results have little clinical value. We hope that the findings of this study may attract the attention of clinicians who use glucocorticoids to treat severe patients, and provide reference for similar clinical studies in the future. Despite this, we emphasize that these results need to be interpreted with caution. Finally, we note that some RCTs [27, 29] have shown the advantages of methylprednisolone, such as increasing PaO_2_/FiO_2_ ratio, shortening hospital stay, and reducing nosocomial infections. Therefore, RCTs with a larger sample size are required to confirm the effectiveness and safety of methylprednisolone in the treatment of patients with severe COVID-19.

## Conclusions

The present meta-analysis showed that compared with dexamethasone, methylprednisolone could significantly reduce the systemic inflammatory response in severe COVID-19 patients, and its effect was equivalent to that of dexamethasone on other clinical outcomes. It should be noted that the heterogeneity across studies was significant, and the equivalent dose of methylprednisolone used was relatively higher. Based on the evidence of subgroup analyses of RCTs, it is believed that methylprednisolone treatment has the potential to improve the prognosis of patients with severe COVID-19, which needs to be verified by high-quality RCTs with a larger sample size.

## Supplementary Information

**Additional file 1.** PRISMA checklist.

**Additional file 2: Supplementary Table 1.** Score of methodological items for non-randomized studies (MINORS). **Supplementary Figure 1.** Summary of risk of bias for randomized controlled trials. **Supplementary Figure 2.** Subgroup analysis of different courses of glucocorticoids treatment for the comparison of short-term mortality. **Supplementary Figure 3.** Subgroup analysis of different doses of methylprednisolone for the comparison of short-term mortality. **Supplementary Figure 4.** Subgroup analysis of different courses of glucocorticoids treatment for the comparison of ICU admission rate. **Supplementary Figure 5.** Subgroup analysis of different courses of glucocorticoids treatment for the comparison of mechanical ventilation rate. **Supplementary Figure 6.** Sensitivity analysis for the comparison of hyperglycemia rate.

## Data Availability

The datasets analyzed during the present study are available from the corresponding author on reasonable request.

## Abbreviations

ARDS: Acute respiratory distress syndrome
CI: Confidence interval
CRP: C-reactive protein
ICU: Intensive care unit
MD: Mean difference
MINORS: Methodological index for non-randomized studies
MERS-CoV: Middle East respiratory syndrome coronavirus
RCT: Randomized controlled trial
RR: Risk ratio;
SARS-CoV-2: Severe acute respiratory syndrome coronavirus 2
WHO: World Health Organization
