# Supplementary material for: A systematic review and meta-analysis of glucocorticoids treatment in severe COVID-19: methylprednisolone versus dexamethasone
Source: BMC Infect Dis. 2023 May 5;23:290. doi: 10.1186/s12879-023-08280-2 (PMC10162003; doi:10.1186/s12879-023-08280-2)
Supplement: Supplementary file 2 — Additional file 2: Supplementary Table 1. Score of methodological items for non-randomized studies (MINORS). Supplementary Figure 1. Summary of risk of bias for randomized controlled trials. Supplementary Figure 2. Subgroup analysis of different courses of glucocorticoids treatment for the comparison of short-term mortality. Supplementary Figure 3. Subgroup analysis of different doses of methylprednisolone for the comparison of short-term mortality. Supplementary Figure 4. Subgroup analysis of different courses of glucocorticoids treatment for the comparison of ICU admission rate. Supplementary Figure 5. Subgroup analysis of different courses of glucocorticoids treatment for the comparison of mechanical ventilation rate. Supplementary Figure 6. Sensitivity analysis for the comparison of hyperglycemia rate. [file 12879_2023_8280_MOESM2_ESM.docx]

| Supplementary Table 1 Score of methodological items for non-randomized studies (MINORS) | | | | | | | | | | | | | |
| --- | --- | --- | --- | --- | --- | --- | --- | --- | --- | --- | --- | --- | --- |
| Study | A clearly stated aim | Inclusion of consecutive patients | Prospective collection of data | Endpoints appropriate to the aim of the study | Unbiased assessment of the study endpoint | Follow-up period appropriate to the aim of the study | Loss to follow up less than 5% | Prospective calculation of the study size | An adequate control group | Contemporary groups | Baseline equivalence of groups | Adequate statistical analyses | Score |
| Fatima, 2020 [18] | 2 | 2 | 2 | 1 | 0 | 1 | 2 | 0 | 2 | 2 | 1 | 2 | 17 |
| Rana, 2020 [19] | 2 | 0 | 0 | 2 | 0 | 2 | 2 | 0 | 2 | 2 | 1 | 2 | 13 |
| Aslam, 2021 [20] | 2 | 0 | 1 | 2 | 0 | 2 | 2 | 0 | 2 | 2 | 1 | 2 | 16 |
| Buso, 2021 [21] | 2 | 2 | 0 | 2 | 0 | 2 | 2 | 0 | 2 | 2 | 1 | 2 | 17 |
| Du Plessis, 2021 [22] | 2 | 2 | 0 | 2 | 0 | 2 | 2 | 0 | 2 | 2 | 0 | 1 | 15 |
| El mezzeoui, 2021 [23] | 2 | 0 | 0 | 2 | 0 | 1 | 2 | 0 | 2 | 2 | 2 | 2 | 15 |
| Ko, 2021 [24] | 2 | 2 | 0 | 2 | 0 | 1 | 2 | 0 | 2 | 2 | 2 | 2 | 17 |
| Mora-Luján, 2021 [25] | 2 | 2 | 0 | 2 | 0 | 2 | 2 | 0 | 2 | 2 | 2 | 2 | 18 |
| Pinzón, 2021 [26] | 2 | 0 | 1 | 2 | 0 | 2 | 2 | 0 | 1 | 0 | 1 | 2 | 13 |
| Note: The items are scored 0 (not reported), 1 (reported but inadequate) or 2 (reported and adequate). | | | | | | | | | | | | | |


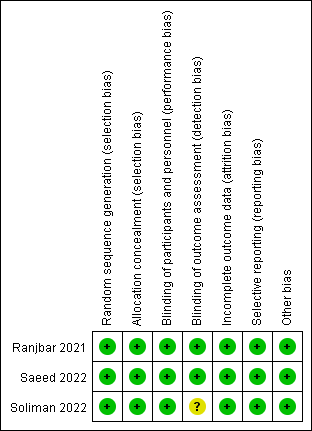


**Supplementary Figure 1:** Summary of risk of bias for randomized controlled trials.


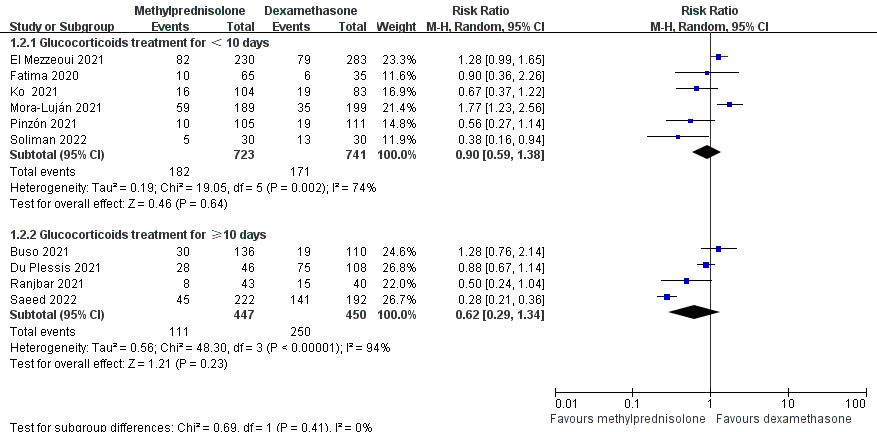


**Supplementary Figure 2:** Subgroup analysis of different courses of glucocorticoids treatment for the comparison of short-term mortality.


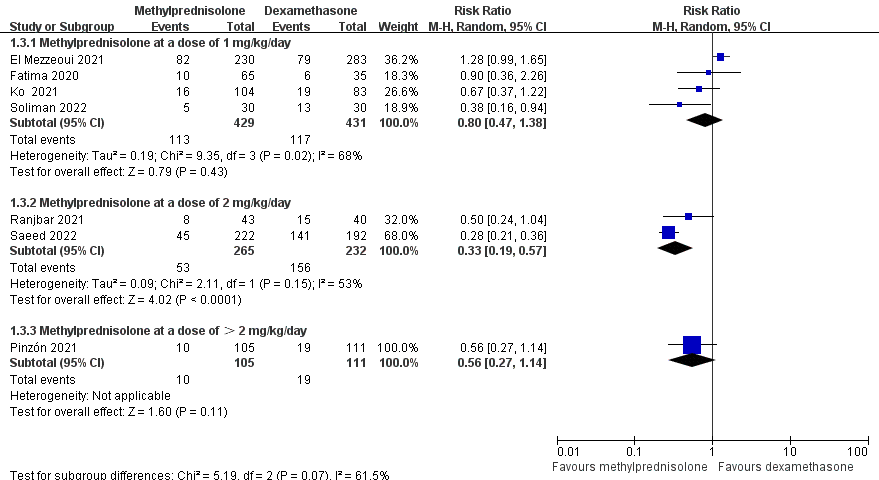


**Supplementary Figure 3:** Subgroup analysis of different doses of methylprednisolone for the comparison of short-term mortality.


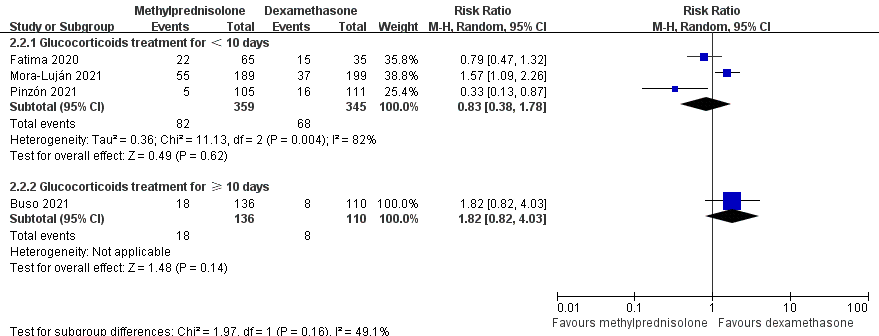


**Supplementary Figure 4:** Subgroup analysis of different courses of glucocorticoids treatment for the comparison of ICU admission rate.


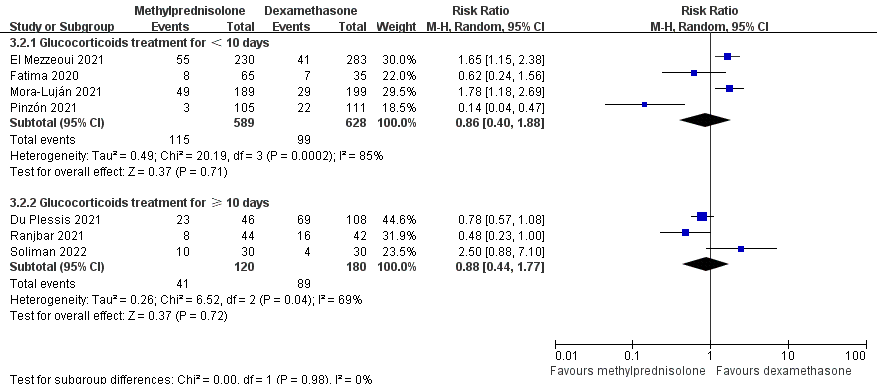


**Supplementary Figure 5:** Subgroup analysis of different courses of glucocorticoids treatment for the comparison of mechanical ventilation rate.


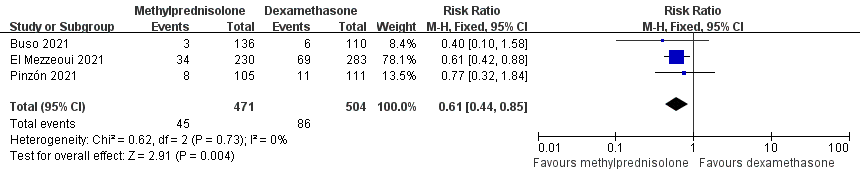


**Supplementary Figure 6:** Sensitivity analysis for the comparison of hyperglycemia rate.
